# Generalized energy failure criterion

**DOI:** 10.1038/srep23359

**Published:** 2016-03-21

**Authors:** R. T. Qu, Z. J. Zhang, P. Zhang, Z. Q. Liu, Z. F. Zhang

**Affiliations:** 1 Shenyang National Laboratory for Materials Science, Institute of Metal Research, Chinese Academy of Sciences, 72 Wenhua Road, Shenyang, 110016, P.R. China

## Abstract

Discovering a generalized criterion that can predict the mechanical failure of various different structural materials is one of ultimate goals for scientists in both material and mechanics communities. Since the first study on the failure criterion of materials by Galileo, about three centuries have passed. Now we eventually find the “generalized energy criterion”, as presented here, which appears to be one universal law for various different kinds of materials. The validity of the energy criterion for quantitatively predicting the failure is experimentally confirmed using a metallic glass. The generalized energy criterion reveals the competition and interaction between shear and cleavage, the two fundamental inherent failure mechanisms, and thus provides new physical insights into the failure prediction of materials and structural components.

For centuries, scientists have made great efforts to develop theories for predicting and controlling the mechanical failure of structural components^1^,^2^. Generally, there are two classes of prediction theories, i.e., fracture mechanics and failure criterion. Fracture mechanics deals with the problem of crack propagation and can well predict the instable fracture of the crack-contained components, and thus is frequently used in industry for assessing the safety of components^2^,^3^. On the other hand, the failure criterion, which predicts the critical conditions for the breakdown of elasticity of materials (i.e., yielding for ductile materials and fracture for brittle materials), is often employed during the structural design stage of a component to ensure it safely work in the elastic state of the used material^1^,^3^.

The gate of fracture mechanics was opened by the pioneering work by Griffith^4^, who firstly applied the first law of thermodynamics to solve the failure problem of a cracked glass, and proposed the critical energy as a criterion to predict the instable fracture of the oxidation glass. Now the energy criterion for fracture has been generally recognized and frequently applied in various materials^2^. Although for different materials the inherent resistance to crack formation and propagation may be different, the energy criterion for cracking can be written in a similar universal formula as,


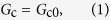


where *G*_c_ is the input energy, and *G*_c0_ is a material property (e.g., toughness) determining the energetic resistance to unstable propagation of crack. For brittle materials like oxidation glass or ceramic, *G*_c0_ only depends on the surface energy; while *G*_c0_ of ductile materials such as copper should also incorporate the energy consumed by plastic deformation ahead of crack tip, as suggested by Orowan^5^ and Irwin^6^.

The failure criterion, which no matter whether is essentially the yield criterion for ductile materials or the fracture criterion for brittle materials that fracture without any plasticity, has a longer history. In the 17^th^ century, when Galileo, the inventor of the concept of stress, firstly employed combined mathematic and experimental methods to study the failure of solid^1^,^3^,^7^, he found that fracture happens along the plane with the maximum normal stress (*σ*_max_), i.e.,


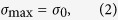


where *σ*_0_ is a constant, meaning the critical normal stress for cleavage fracture. This criterion is now called as the maximum normal stress criterion (or the Rankine criterion), which can well predict the fracture of various brittle materials with failure dominated by crack formation. In the 19^th^ century, Tresca^1^ investigated a lot on the plastic deformation of metals, and claimed that the flow of metals often happens along the plane with the maximum shear stress (*τ*_max_), i.e.,


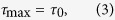


where τ_0_ is the critical shear stress for yielding. This criterion is now termed as the maximum shear stress criterion (or the Tresca criterion), and can be widely used to predict the yield conditions of those ductile metals. Later Maxwell, Huber and von Mises^3^,^8^ also proposed a criterion dominated by the deviatoric stresses (now named as the von-Mises criterion) to predict the yielding conditions of metals and alloys.

Although fracture mechanics and failure criterion play vital roles in different stages of the safety design of structural components, a comparison of these two kinds of prediction theories may provide some inspirations. Note that the von-Mises criterion is essentially an energy criterion with the maximum distortional energy density as the critical condition^3^, i.e.,


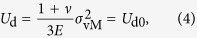


where *ν* is Poisson’s ratio, *E* is Young’s modulus, *σ*_vM_ is the equivalent von-Mises stress, *U*_d_ is distortional energy density, and *U*_d0_ is a constant.

Moreover, both of the Rankine and the Tresca criteria predict the failure that corresponds to the elasticity breakdown occurring at some critical states. The critical states were originally characterized by the critical stresses (*σ*_0_ and *τ*_0_), as illustrated in Fig. 1. However, at the critical state, besides the stress, the elastic energy density also reaches the critical value, i.e., the critical cleavage energy density


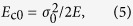


and the critical shear energy density


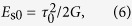


where *G* is shear modulus. Here the linear elastic behavior is assumed.

Because the cleavage fracture needs energy to break the atomic bonding to form the crack and the initiation of shear deformation also requires energy to activate the plastic unit like dislocation, the Rankine and Tresca criteria may be substantially also energy criteria. Since the failure of materials (yielding or fracture) often originates from a specific plane (e.g., slip plane, shear band plane or cracking plane, etc.), the shearing along this plane driven by the shear stress (*τ*) and the cleavage for separating the plane driven by the normal stress (*σ*) should be the two basic failure mechanisms, as shown in Fig. 1. Thus we can respectively define two energy densities associated with the failure plane: i.e., the cleavage energy density,


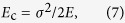


and the shear energy density,


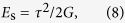


Consequently, the energy forms of the Rankine criterion and Tresca criterion are,


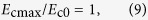


and


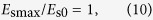


respectively. Equation (9) tells that material fractures at the plane with the maximum cleavage energy density, while the Tresca criterion (Eq. (10)) indicates that material yields at the plane with the maximum shear energy density.

## Results

The energy basis of the above failure criteria and the energy criterion for crack propagation suggest that the failure of materials may be generally dominated by energy. It is also worth noting that the materials that fail according to the Tresca criterion are mostly very ductile^1^,^3^. This indicates that the energy barrier for shear deformation (*E*_s0_) should be very low while that for cleavage fracture (*E*_c0_) must be high. On the other hand, the materials that can be predicted by the Rankine criterion are usually quite brittle^1^,^3^, implying a low *E*_c0_ but high *E*_s0_. Obviously, either the Rankine or the Tresca criterion considers only one of the two basic failure mechanisms (cleavage or shear, respectively), which leads to the lack of general applicability. Therefore, to predict various different materials, it is necessary to consider both the shear and cleavage mechanisms for the failure, and the following generalized energy criterion may be proposed:


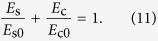


Note here *E*_c0_ and *E*_s0_ are two energy barriers that represent the critical energies required for a unit volume of material cleaving under pure tension and failing in simple shear, respectively. Eq. (11) suggests that the failure of materials is caused by the synergic effects of both shear and cleavage associated with the most dangerous plane. Thereby, the ratio of 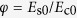 can be defined as a material constant determining the inherent failure mechanism of material. As one can see, if a material shows that *φ* ≫ 1, the cleavage fracture will be much easier to occur than the shear failure; hence the failure of this material must be intrinsically brittle and dominated by cleavage fracture. In this case, Eq. (11) can be approximated as 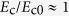, which is the form of the Rankine criterion. On the contrary, if a material exhibits that *φ* ≪ 1, its failure should be dominated by shear, and Eq. (11) evolves to 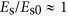, similar to the Tresca criterion. Therefore, the energy criterion of Eq. (11) may unify the classical Rankine and Tresca criteria and should be a more general failure criterion, which is also expected to predict the failure of materials with comparable energy barriers for shear and cleavage, such as metallic glasses (MGs).

**Figure 2:**
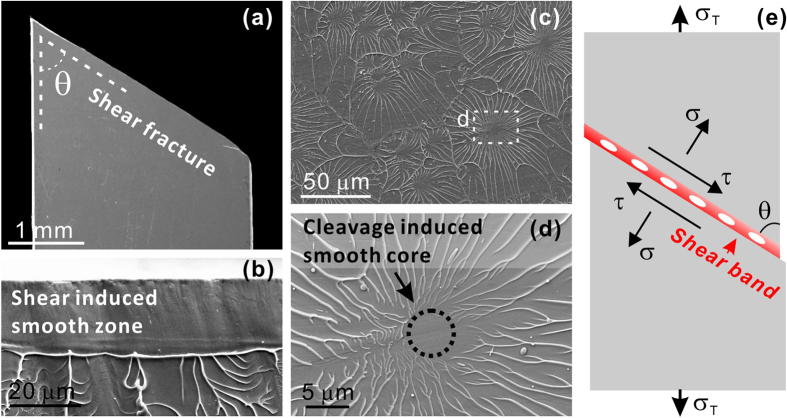
Tensile fracture features and failure mechanism of a typical Zr-based MG. (**a**) Tensile fracture appearance showing fracture along an angle of *θ* with respect to the loading direction (vertical). (**b**–**d**) Typical fracture features on the fracture surface: (**b**) smooth zone induced by shear, (**c**,**d**) radiating vein patterns with cleavage induced smooth cores. (**e**) Illustration of tensile failure mechanism including shear band deformation and micro-scale cleavage fracture.

Owing to the amorphous structure free from crystalline defects, MGs exhibit promising properties such as extremely high strength^9^. Under unconfined loading like uniaxial tension, MGs usually show near-zero ductility and catastrophic fracture behavior^10^, akin to brittle materials; while inside one shear band, which is the media for plastic deformation at room temperature, the plastic strain to fracture can be quite great^11^. These unique behaviors imply that the energy barriers for shear and cleavage failures for MGs may be comparable. We can further examine the competition between shear and cleavage in MGs by analyzing the tensile fracture mechanism. As shown in Fig. 2(a), typical Zr-based MGs fracture in a shear-dominated mode along the major shear band under tension. Besides the smooth zone produced by plastic shearing^10^ (Fig. 2(b)), the fracture morphology also consists of plenty of smooth cores that are pointed to by tips of radiating veins, as dotted by circles in Fig. 2(c,d), which is manifested as the micro-scale cleavage cracks inside the major shear band prior to fracture^10^,^12^,^13^,^14^. The fracture features displayed in Fig. 2 have been widely observed in many other MG systems^14^,^15^,^16^,^17^, indicating those are common behaviors of MGs (not including brittle MGs without shear banding). Therefore, the tensile failure mechanism of MGs essentially includes both the shear deformation and cleavage fracture, which implies that their failure may be predictable by the proposed energy criterion.

**Figure 3:**
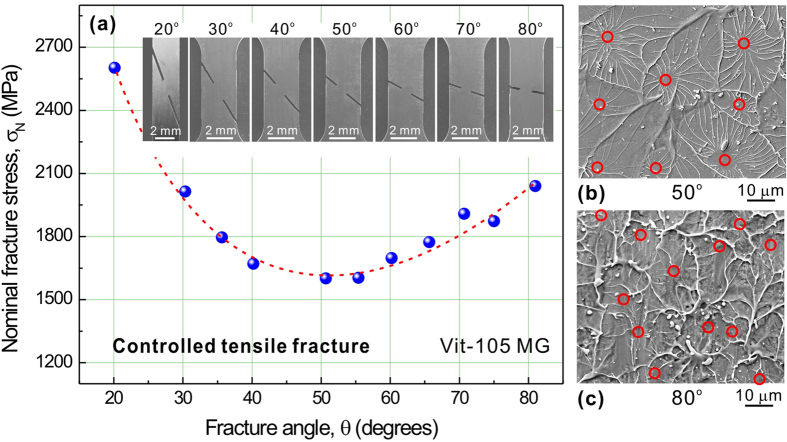
Experimental results of controlled tensile fracture of Vit-105 MG^10^,^18^. (**a**) Nominal fracture stress (*σ*_N_) as a variation of fracture angle (*θ*). 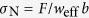, where *F* is the maximum force at fracture, *w*_eff_ is the width between two notch tip and *b* is the thickness. Since fracture occurs along the designed fracture plane, the fracture angle takes the value of the measured notch angle. (**b**,**c**) Typical tensile vein pattern on the fracture surface with dotted circles indicating the local cleavage zones: (**b**) 50° sample, (**c**) 80° sample.

To examine the validation of the energy criterion for predicting the failure of MGs, a series of fracture-controlled tensile tests were conducted using Vit-105 MG^18^. In these tests, tensile fracture was controlled to occur along different planes by introducing inclined notches to dog-bone specimens, as shown in the inset of Fig. 3. More experimental details can be found in Supplementary Material. The results of uniaxial tension show that Vit-105 MG fractures at a strength of *σ*_T_ = 1660 MPa and along an intrinsic fracture angle^18^ of *θ*_T_ = 50.7°. Thus the total strain energy density for fracture should be 
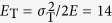
 MJ/m^3^, with the Young’s modulus *E* = 97.8 GPa^19^. Figure 3(a) shows the nominal fracture stress (*σ*_N_) as a variation of fracture angle. As increasing fracture angle from 20° to 80°, *σ*_N_ firstly decreases and then increases, showing a minimum at the angle of ~50°, very close to *θ*_T_ = 50.7°. The measured nominal strength at the angle of ~50° is ~1601 MPa, which is also close to the fracture strength of smooth specimen (~1660 MPa). This means that fracture along the plane with or without inclined notches requires nearly the same applied stress. In other words, the introduced notches have little influences on the measured failure stresses of the fracture planes. Additional analysis (see Supplementary Material) demonstrates that the nominal fracture stresses in Fig. 3(a) should rationally reflect the different fracture resistance of different plane in a Vit-105 MG tensile specimen, i.e., the plane with the angle of *θ*_T_ is the easiest one to break. Why does the fracture of this MG behave like this? Can the energy criterion predict? To find the answers, let us firstly calculate the shear and cleavage energy densities associated with the fracture plane in each specimen. Based on the above analysis, the shear and normal stresses that control the fracture can be written as:


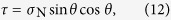



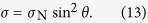


Substituting Eqs (12, 13) into Eqs (7, 8), we finally get the shear and cleavage energy densities associated with the failure plane with the angle *θ*:


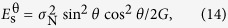



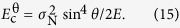


It is easy to find that if MG fractures along the plane with an angle higher than *θ*_T_, the contribution by cleavage to failure should be larger. Observations on the fracture morphologies of the inclined notch tensile specimens exhibit an increased density but a similar size of local cleavage zone with increasing fracture angle, as shown in Fig. 3(b,c), which confirms the increased fraction of cleavage fracture.

**Figure 4:**
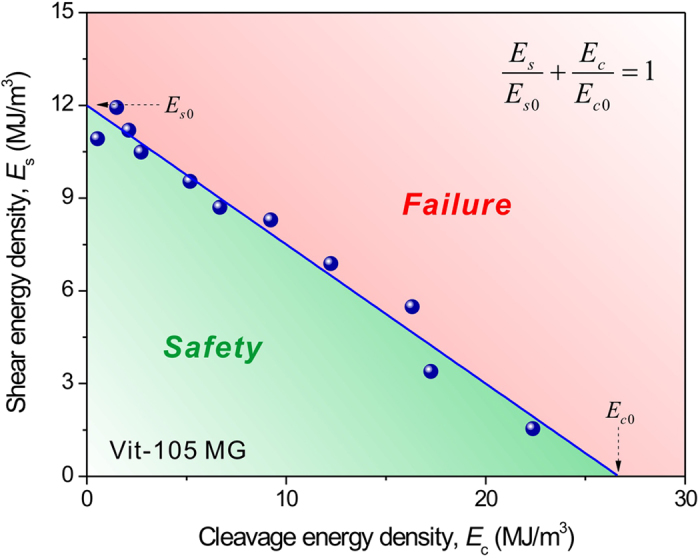
Energy diagram for the tensile failure of Vit-105 MG. The red dots from experimental results determine a critical failure line of energy density, below which the MG should be always safe.

The fracture-controlled experiment provides a series of failure conditions with varying energy densities; hence the critical failure line of energy density can be determined. Figure 4 plots the *E*_s_ vs. *E*_c_ of tensile specimens fractured along different planes. A linear relationship between *E*_s_ and *E*_c_ can be obviously seen, in remarkably good accordance with the proposed generalized energy criterion (Eq. (11)). The fitted two constants *E*_s0_ = 12 MJ/m^3^ and *E*_c0_ = 27 MJ/m^3^, and the ratio 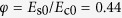, indicating that the failure of Vit-105 MG is dominated by shear (e.g., shear banding) and also strongly influenced by cleavage. This result confirms the validity of the generalized energy criterion for predicting the tensile fracture of Vit-105 MG. Further analysis on the two energy densities associated with the fracture plane of uniaxial tensile specimen (*θ*_T_ = 50.7°) shows that *E*_s_ = 9.2 MJ/m^3^, and *E*_c_ = 5.0 MJ/m^3^, both of which are smaller than the two constants, *E*_s0_ and *E*_c0_, respectively, demonstrating that both the Rankine and Tresca criteria are inappropriate for predicting the fracture of the Vit-105 MG.

The energy criterion can be also assessed by examining its prediction of the fracture angle under uniaxial tension. Note that the specimen fractures under uniaxial tension along the *θ*_T_ plane, which is neither the plane with the maximum shear energy density (*θ* = 45°) nor the plane with the maximum cleavage energy density (*θ* = 90°). To predict the fracture angle with the energy criterion, we calculate the totally input energy density (*E*_T_) as the function of *θ*. According to Eqs (14, 15), the two energy densities associated with the fracture plane with angle of *θ* can be written as,


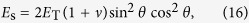



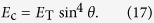


Substituting Eqs (16, 17) into the energy criterion (Eq. (11)) and rearranging the equation, we obtain


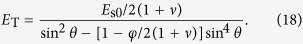


For most MGs, *φ* < 1; hence *E*_T_ has a minimum at a definite angle that should correspond to *θ*_T_, i.e.,


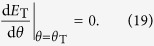


Thus,


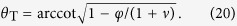


And the minimum strain energy density will be,


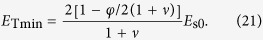


Substituting *φ* = 0.44, *E*_s0_ = 12 MJ/m^3^ (see Fig. 4) and *ν* = 0.37 (reference^20^) for Vit-105 MG into Eqs (16, 17), one calculates the *θ*_T_ = 50.6° and *E*_Tmin_ = 14.7 MJ/m^3^, in good agreement with the measured values of the smooth specimen. This also answers the raised question above: MG fails under tension always along an intrinsic fracture plane with the angle of *θ*_T_ because this requires the minimum input energy.

## Discussion

Like the Tresca criterion and the Rankine criterion, the present energy criterion can also be written in the form of stresses. Substituting Eqs (5, 6, 7, 8) into Eq. (11), we get,


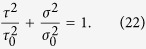


This equation is the same as the Ellipse criterion that was proposed by Zhang and Eckert^21^ to be a unified tensile fracture criterion. By varying the fracture mode factor of 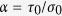, the Ellipse criterion can unify the classical failure criteria and predict the tensile failure of a broad of materials. Obviously, the present energy criterion provides a physical interpretation for the Ellipse criterion from the energetic perspective, further clarifying the synergic effects of two basic mechanisms of shear and cleavage on the failure of materials.

### Application for predicting the failure

As a failure criterion, the generalized energy criterion can be employed to predict the critical conditions for failure of materials or structural component at complex stress states. Practically, the present criterion considers the most dangerous plane where the failure will happen. In order to apply the criterion, we need to determine what plane is the most dangerous. From the energy consideration, the failure should happen with the minimum total input energy, which should be the principle to find the most dangerous plane. According to the material type, when applying the present criterion, there are two cases.

1. For isotropic materials (e.g., MGs), any plane in the material has the same properties as other ones and thus may probably be the final failure plane. In this case, we can apply the criterion to predict the failure conditions according to the following procedure. (*a*) *Determine the two energy barriers for failure* (*E*_*s0*_ *and E*_*c0*_). Usually, if the material is ductile, plastic yielding may happen before fracture, so the yield stress in the simple shear test should be the *τ*_0_ and thus the *E*_s0_ can be determined. Under uniaxial tension, the material also tends to yield first; thus the elastic strain energy density for yielding under uniaxial tension, *E*_Ty_, should not be the energy barrier for cleavage fracture, *E*_c0_. However, since the failure happens with the minimum energy input, we can make the measured *E*_Ty_ equal to the *E*_Tmin_ predicted by the present energy criterion according to Eq. (21), then *E*_c0_ can be obtained. On the other hand, if the material is brittle and cleavage fracture occurs before yielding, the elastic strain energy density for fracture under uniaxial tension should be the energy barrier for cleavage fracture, i.e., *E*_c0_ = *E*_Tf_. However, for brittle materials even under simple shear, the fracture may still be controlled by cleavage fracture, thus the *E*_s0_ cannot be directly measured by simple shear test. In this case, double shear test can be done. By introducing shear stress in another direction, the fracture mode will become a mixed mode of shear and cleavage. Through measuring the fracture angle and fracture stress, the *E*_s0_ can be finally determined. (*b*) *Assuming the failure occurs along the plane with the angle of θ, calculate the stresses* (*σ*_*θ*_*, τ*_*θ*_) *on this plane and the associated energy density* (*E*_*cθ*_*, E*_*sθ*_). For example, for the stress state of uniaxial tension, we can directly use Eqs (12, 13, 14, 15, 16, 17) to calculate the stresses and energy densities, while for other cases a careful stress analysis may be required. (*c*) *Apply the energy criterion with above parameters and derive the equation for the input total energy* (*E*_*t*_) *as a function of angle θ*. For tension, Eq. (18) can thus be obtained. (*d*) *Calculate the minimum of the total input energy and the corresponding θ, which should be the fracture conditions predicted by the present energy criterion.* For instance, Eqs (20, 21) are the predicted results for failure of isotropic materials under tension.
2. For anisotropic materials (e.g., single crystals, fiber-reinforced composites, laminates, etc.), different planes in the materials may have different properties. To apply the present criterion, we need firstly to find all kinds of planes, and then determine the two energy barriers for failure (*E*_s0_ and *E*_c0_) of each plane. Since the anisotropy, some special designed experiments may be required, which can be referred to the studies on the failure criterion of composite materials^3^. Similar to the above procedure, the shear and normal stresses on the possible planes require to be calculated by stress analysis, and then the associated energy density can be obtained. The energy criterion will be applied for all kinds of planes, while the failure conditions (including the failure plane and the failure stress) should correspond to the plane that failure occurs requiring the minimum total input energy. The application of the energy criterion to anisotropic materials still requires further investigations, including experimental validations.

### Establishment of the intrinsic failure mechanism map

As discussed above, the present energy criterion includes the contributions from both of the two basic failure mechanisms, thus is expected to predict the failure of a broad range of materials from ductile to brittle. The material constant 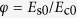, which determines the intrinsic failure mechanism, i.e., shear controlled or cleavage controlled failure, can be correlated to *α* by,


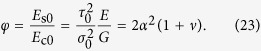


**Figure 5:**
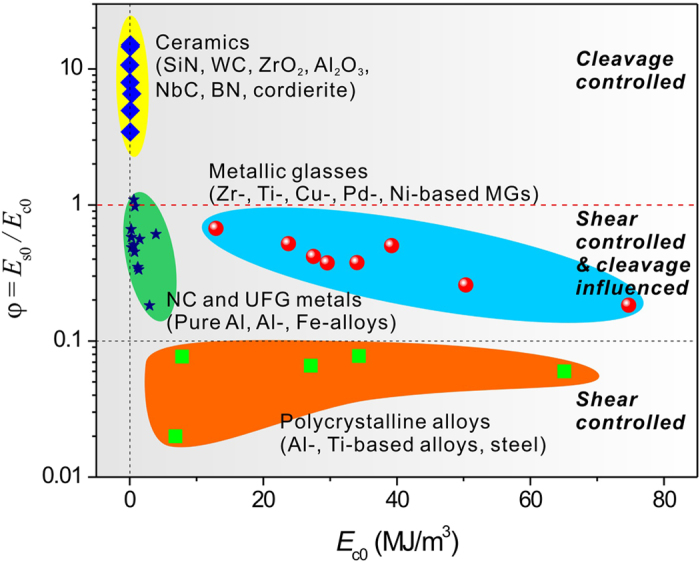
Intrinsic failure mechanism map of materials. Data points were plotted based on data in Supplementary Tables S1–S3. Note that the outline of each material zone is not the accurate boundary of the corresponding material kind, only embracing the current data points and suggesting a trend.

It has been found that the factor *α* reflects the effect of normal stress on the failure and strongly correlates to the strength ratio between compression and tension in isotropic materials^21^,^22^. Consequently, if the tensile and compressive failure strengths and elastic moduli of an isotropic material are known, the two energy barriers for shear and cleavage failures, *E*_s0_ and *E*_c0_, as well as the constant *φ* can be obtained (see more discussions in supplementary material). We collected the strength and modulus data of some MGs, nanocrystalline (NC) and ultra-fine grained (UFG) metals, polycrystalline alloys and ceramics, and estimated the critical energy densities, as listed in Supplementary Tables S1–S3. Based on the data available, the intrinsic failure mechanism map of materials may be plotted in Fig. 5. It can be seen that ceramics show very low *E*_c0_ and very high *φ*, which means that the cleavage fracture in these brittle materials are quite easy to occur while the shear failure should be rather difficult. On the other hand, for polycrystalline alloys, although *E*_c0_ varies in a wide range due to their different composition and structure, the energy density ratio, *φ*, is rather low, namely smaller than 0.1, demonstrating that the shear deformation (mainly via dislocation slipping) should be the controlled failure mechanism. For NC, UFG and MG materials, although *E*_s0_ is still smaller than *E*_c0_ that means the shear mechanism controls the failure, the ratio *φ* is considerably higher than that for polycrystalline alloys, suggesting that the cleavage mechanism must have significant influence on the final failure. These predictions are well consistent with the experimental observations^23^,^24^,^25^,^26^.

In conclusion, this work proposes a generalized energy criterion for predicting the failure of materials, which is particularly applicable for the materials with comparable energy barriers for shear and cleavage, such as MGs, NC and UFG materials^23^,^24^,^25^,^26^,^27^,^28^,^29^. Quantitative experiment confirms the validity of the proposed generalized energy criterion for predicting the tensile failure of MG. Based on the energy criterion, the intrinsic failure mechanism map of materials is quantitatively established, which explicitly gives an exhibition and also a prediction of the failure mode of each kind of materials. With a simple linear relation, the generalized energy criterion illustrates materials’ failure as an energetic competition between the two basic failure mechanisms: shear and cleavage, which may suggest a new understanding on the intrinsic toughness of materials. The generalized energy criterion is consistent with the classical approaches of mechanics for predicting failure via energy methods, but provides new physical insights into the failure criterion by considering the inherent failure mechanism of materials.

## Methods

We selected an MG with a composition of Zr_52.5_Cu_17.9_Ni_14.6_Al_10_Ti_5_ (at.%) (labeled as Vit-105 MG) and conducted a series of fracture-controlled tensile tests. The Vit-105 MG plates with a size of 40 × 30 × 1.8 mm^3^ were prepared by copper mold casting in a high-purity argon atmosphere. The amorphous structure of the specimens was identified by standard X-ray diffraction. In the fracture-controlled tensile tests, fracture was controlled to occur along the designed planes with different angles with respect to the loading axis, by means of introducing inclined notches to dog-bone specimens. The notch angle (*θ*_n_) was accurately controlled and varied from 20° to 80° with respect to the loading axis. All specimens have a thickness of ~1.5 mm and gauge width of ~3 mm. The gauge length for notched specimens with notch angle from 30° to 80° is ~7 mm, while it is ~10 mm for 20° specimen to ensure the accurate notch angle. All notches are U-shaped and have a notch width of ~0.2 mm and a notch tip radius of ~0.1 mm. Smooth specimens without notches were also prepared to measure the tensile strength and observe the fracture features. Tensile tests were conducted with an MTS 810 testing machine at a strain rate of 10^−4 ^s^−1^ at room temperature. The fracture features were observed with a Leo Supra 35 scanning electron microscope (SEM). By introducing the inclined notches with angle of *θ*_n_ and properly designing the width of notched region, the area of the notch plane is controlled to be smaller than or equal to (only when *θ*_n_ *=* *θ*_T_) that of the intrinsic tensile fracture plane (i.e., the plane with the angle of *θ*_T_). Hence the fractures of inclined notch tensile specimens were well controlled and occurred along the designed notch planes (see Fig. 7 in ref. 18).

## Additional Information

**How to cite this article**: Qu, R. T. *et al.* Generalized energy failure criterion. *Sci. Rep.* **6**, 23359; doi: 10.1038/srep23359 (2016).

## Supplementary Material

Supplementary Information

## Figures and Tables

**Figure 1:**
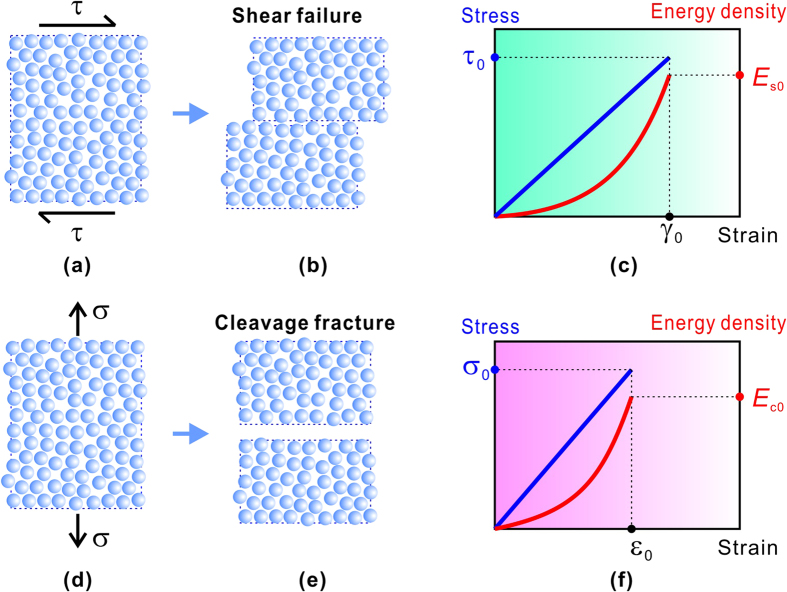
Illustrations on the two basic failure mechanisms of material. (**a**–**c**) shear failure, (**d**–**f**) cleavage fracture.
