# Supplementary material for: Generalized energy failure criterion
Source: Sci Rep. 2016 Mar 21;6:23359. doi: 10.1038/srep23359 (PMC4800311; doi:10.1038/srep23359)
Supplement: Supplementary Information [file srep23359-s1.doc]

**SUPPLEMENTARY DISSCUSSIONS, DATA AND REFERENCES**

**Generalized Energy Failure Criterion**

R. T. Qu^*^, Z. J. Zhang, P. Zhang, Z. Q. Liu, Z. F. Zhang^*^

Shenyang National Laboratory for Materials Science, Institute of Metal Research,

Chinese Academy of Sciences, 72 Wenhua Road, Shenyang, 110016, P.R. China

^*^**Correspondence** should be addressed to R.T.Q. (rtqu@imr.ac.cn) and Z.F.Z. (zhfzhang@imr.ac.cn)

**1. Additional discussions**

*1.1 Fracture strength of notched MGs*

The measured nominal strength at the angle of ~50° is ~1601 MPa, which is also close to the fracture strength of smooth specimen (~1660 MPa) [S1]. This means that fracture along the plane with or without inclined notches requires nearly the same applied stress. In other words, the introduced notches have little influences on the measured failure stresses of the fracture planes.

In fact, notch tensile experiments by Qu *et al.* [S2] and MD simulations by Sha *et al.* [S3] showed that MGs are quite distinctive from traditional brittle materials, in which notch has great influence on fracture strength, while the strength of MGs behaves insensitive to notch. Furthermore, finite element analysis shows that the stress concentration zones ahead of the notch tips locate in very small regions comparing with the effective width [S1]. The stress concentration zone will influence the initiation of shear band but should have weak impact on the shear band propagation. It has been proved that the propagation of shear band requires that all points on the entire propagation path reach a critical stress, rather than to be determined by the locally maximum stress [S4]. Accordingly, the fracture conditions of the inclined notch tensile samples should be determined by the minimum stress at the fracture plane (i.e., approximately to be the nominal stress), rather than the maximum stress at the notch tip.

*1.2 Equations for the critical failure strengths and critical energy densities*

As discussed in the article, it is easy to directly deduce the Ellipse criterion from the generalized energy criterion, which means that the two criteria are essentially equivalent. The Ellipse criterion emphasizes that the normal stress acting on the shear plane affects the failure behavior and failure conditions, which has been supported by extensive experimental observations [S1,S5,S6,S8,S14,S17]. It is also found that the effects of normal stress are different between compressive and tensile stress states [S1], which leads to development of the universal failure criterion [S5]. The universal failure criterion has a same equation with the Ellipse criterion under tensile stress states (i.e., $\sigma_{1}+\sigma_{3}\geq0$, with *σ*_1_ and *σ*_3_ are the maximum and minimum principal stresses, respectively), i.e., $\tau^{2}/\tau_{0}^{2}+\sigma^{2}/\sigma_{0}^{2}=1$. However, under compressive stress states (i.e., $\sigma_{1}+\sigma_{3}<0$), the universal failure criterion becomes $\tau^{2}/\tau_{0}^{2}+\beta\sigma^{2}/\sigma_{0}^{2}=1$, with *β* <0 an extrinsic parameter representing the inverse and weak normal stress effect comparing with that under tension. Obviously, similar with the Ellipse criterion, the energy form of the universal failure criterion should also give a linear relation between normalized shear energy density and normalized cleavage energy density, but the contribution by cleavage energy works totally differently between compression and tension, which can be easily understood. Because the negative normal stress induced negative cleavage energy under compressive stress states tends to close the cleavage crack and also increases the resistance of shearing, the term of cleavage energy in the energy criterion under compression should be negative. This suggests that the generalized energy criterion and the universal failure criterion should be also essentially equivalent. Based on the universal failure criterion, we can thus calculate the critical shear strength and critical cleavage strength when only knowing the uniaxial tensile and compressive failure strength, which then makes it easy to obtain the critical energy densities for shear and cleavage of materials. It has been experimentally found that for MGs, *β* = -1/2, is an almost constant value. Actually, if varying *β* from -0.5 to -1, only a change of 7%-16% for the value of the fracture mode factor *α*, when the ratio of compressive to tensile strengths ($R=\sigma_{cf}/\sigma_{tf}$) varies in a much wide range from 1.0 to 30. Therefore, for the purpose of simply comparing the different intrinsic failure mechanism of different kind of materials, it is rational to approximately assume that *β*= -1/2 is a constant. In the following, we shall firstly present the equations and then calculate the critical failure strengths and energy densities for various materials. The data will be listed in Tables S1-S3.

According to the universal failure criterion [S5], the fracture mode factor *α* is correlated with the ratio of compressive to tensile strengths ($R=\sigma_{cf}/\sigma_{tf}$), i.e.,

$\alpha=\sqrt{\frac{R^{2}-1}{R^{2}+1/2}}$, when $1\leq R\leq\sqrt{10}/2$ (S1)

$\alpha=\sqrt{\sqrt{1+R^{2}/2}-1}$, when $R>\sqrt{10}/2$ (S2)

For materials with strength ratio $R>\sqrt{10}/2$, the fracture mode factor $\alpha>\sqrt{2}/2$, and they often fracture in cleavage mode under tension. Hence the critical cleavage and shear strengths are,

$\sigma_{0}=\sigma_{tf}$, (S3)

and

$\tau_{0}=\alpha\sigma_{0}=\sigma_{tf}\sqrt{\sqrt{1+R^{2}/2}-1}$, (S4)

respectively. And the critical cleavage and shear energy densities are

$E_{c0}=\sigma_{0}^{2}/2E=\sigma_{tf}^{2}/2E$, (S5)

and

$E_{s0}=\tau_{0}^{2}/2G=\sigma_{tf}^{2}(\sqrt{1+R^{2}/2}-1)/2G$, (S6)

respectively. Thus the energy density ratio will be

. (S7)

On the other hand, for materials with strength ratio in the range of $1\leq R\leq\sqrt{10}/2$, the fracture mode factor will be in the range of $0\leq\alpha\leq\sqrt{2}/2$. In this case, materials usually fail in a shear mode under either tension or compression. Thus the critical shear and critical strengths can be calculated according to the failure criterion as,

$\tau_{0}=\frac{\sigma_{tf}}{2\sqrt{1-\alpha^{2}}}=\frac{\sigma_{tf}}{2\sqrt{3}}\sqrt{2R^{2}+1}$, (S8)

and

$\sigma_{0}=\tau_{0}/\alpha=\frac{\sigma_{tf}(2R^{2}+1)}{2\sqrt{6(R^{2}-1)}}$, (S9)

respectively. And the critical shear and cleavage energy densities are

$E_{s0}=\tau_{0}^{2}/2G=\frac{\sigma_{tf}^{2}}{24G}(2R^{2}+1)$, (S10)

and

$E_{c0}=\sigma_{0}^{2}/2E=\frac{\sigma_{tf}^{2}(2R^{2}+1)^{2}}{48E(R^{2}-1)}$, (S11)

respectively. Thus the energy density ratio for these materials can be written as,

. (S12)

Based on Eqs. (S1)-(S12), we thus can calculate the critical failure strengths, critical failure energy densities and two material constants of *α* and *φ*, as long as knowing the strengths under uniaxial loadings and elastic moduli *E* and *G*.

**2. Supplementary Tables**

**Table S1.** Tensile and compressive failure strengths (*σ*_tf_ and *σ*_cf_), elastic modulus (*ν, E, G*), critical failure strengths (*τ*_0_ and *σ*_0_), and critical energy densities for failure (*E*_c0_ and *E*_s0_) of some metallic glasses. Data of failure strengths and elastic modulus are collected from Refs. [S1, S5-S14]. The critical failure strengths and their ratio (*α*) are calculated according to Eqs. (S1), (S8) and (S9). The critical energy densities and their ratio are calculated according to Eqs.(S10)-(S12).

| **Compositions** | ***σ*_cf_ (GPa)** | ***σ*_tf_ (GPa)** | ***σ*_cf_/*σ*_tf_** | ***α* = *τ*_0_/*σ*_0_** | ***τ*_0_**  **(GPa)** | ***σ*_0_**  **(GPa)** | ***ν*** | ***E***  **(GPa)** | ***G***  **(GPa)** | ***φ*** | ***E*_c0_**  **(MJ/m^3^)** | ***E*_s0_**  **(MJ/m^3^)** |
| --- | --- | --- | --- | --- | --- | --- | --- | --- | --- | --- | --- | --- |
| Zr_41.25_Ti_13.75_Ni_10_Cu_12.5_Be_22.5_ | 2.05 | 1.95 | 1.05 | 0.26 | 1.01 | 3.88 | 0.356 | 101.0 | 37.2 | 0.18 | 74.70 | 13.70 |
| Cu_60_Zr_30_Ti_10_ | 2.15 | 2.00 | 1.08 | 0.31 | 1.05 | 3.39 | 0.342 | 114.0 | 42.5 | 0.26 | 50.32 | 12.98 |
| Zr_52.5_Ni_14.6_Al_10_Cu_17.9_Ti_5_ | 1.84 | 1.66 | 1.11 | 0.37 | 0.89 | 2.41 | 0.370 | 97.8 | 35.7 | 0.38 | 29.58 | 11.10 |
| Zr_48_Cu_45_Al_7_ | 1.90 | 1.71 | 1.11 | 0.37 | 0.92 | 2.49 | 0.377 | 90.9 | 33.0 | 0.38 | 34.01 | 12.82 |
| Zr_64.13_Cu_15.75_Ni_10.12_Al_10_ | 1.69 | 1.50 | 1.13 | 0.39 | 0.81 | 2.08 | 0.377 | 78.4 | 28.5 | 0.42 | 27.51 | 11.52 |
| Ni_53_Nb_20_Ti_10_Zr_8_Co_6_Cu_3_ | 3.00 | 2.60 | 1.15 | 0.43 | 1.44 | 3.35 | 0.360 | 143.0 | 52.6 | 0.50 | 39.21 | 19.72 |
| Ti_40_Zr_25_Ni_3_Cu_12_Be_20_ | 1.84 | 1.68 | 1.10 | 0.44 | 0.93 | 2.11 | 0.355 | 94.0 | 34.7 | 0.52 | 23.76 | 12.46 |
| Pd_40_Ni_40_P_20_ | 1.78 | 1.46 | 1.22 | 0.49 | 0.84 | 1.71 | 0.400 | 113.7 | 40.6 | 0.67 | 12.93 | 8.69 |

**Table S2.** Tensile and compressive failure strengths (*σ*_tf_ and *σ*_cf_), elastic modulus (*ν, E, G*), critical failure strengths (*τ*_0_ and *σ*_0_), and critical energy densities for failure (*E*_c0_ and *E*_s0_) of some nanocrystalline (NC) and ultra-fine grained (UFG) metals and alloys with varying grain size. Data of failure strengths and elastic modulus are collected from Refs. [S15-S17,S19-S20]. The elastic modulus for Al–10Ti–2Cu alloy was estimated based on an approach considering both iso-stress and iso-strain conditions [S18]. The critical failure strengths and their ratio (*α*) are calculated according to Eqs. (S1), (S8) and (S9). The critical energy densities and their ratio are calculated according to Eqs.(S10)-(S12).

| **Compositions** | ***σ*_cf_ (GPa)** | ***σ*_tf_ (GPa)** | ***σ*_cf_/*σ*_tf_** | ***α* = *τ*_0_/*σ*_0_** | ***τ*_0_**  **(GPa)** | ***σ*_0_**  **(GPa)** | ***ν*** | ***E***  **(GPa)** | ***G***  **(GPa)** | ***φ*** | ***E*_c0_**  **(MJ/m^3^)** | ***E*_s0_**  **(MJ/m^3^)** |
| --- | --- | --- | --- | --- | --- | --- | --- | --- | --- | --- | --- | --- |
| Fe-10%Cu | 0.72 | 0.53 | 1.37 | 0.61 | 0.33 | 0.54 | 0.32 | 192 | 73 | 0.98 | 0.77 | 0.75 |
| Fe-10%Cu | 0.69 | 0.48 | 1.43 | 0.64 | 0.32 | 0.49 | 0.32 | 192 | 73 | 1.09 | 0.63 | 0.68 |
| Pure Al | 0.19 | 0.15 | 1.22 | 0.49 | 0.09 | 0.18 | 0.36 | 68 | 25 | 0.66 | 0.23 | 0.15 |
| Pure Al | 0.18 | 0.16 | 1.15 | 0.42 | 0.09 | 0.21 | 0.36 | 68 | 25 | 0.49 | 0.31 | 0.15 |
| Pure Al | 0.18 | 0.15 | 1.19 | 0.46 | 0.09 | 0.18 | 0.36 | 68 | 25 | 0.58 | 0.25 | 0.14 |
| Pure Al | 0.06 | 0.06 | 0.98 | 0.00 | 0.03 | - | 0.36 | 68 | 25 | 0.00 | - | 0.02 |
| Al–10Ti–2Cu | 0.34 | 0.31 | 1.10 | 0.35 | 0.17 | 0.47 | 0.35 | 80 | 30 | 0.33 | 1.38 | 0.45 |
| Al–10Ti–2Cu | 0.33 | 0.29 | 1.11 | 0.36 | 0.16 | 0.44 | 0.35 | 80 | 30 | 0.35 | 1.21 | 0.42 |
| Al–10Ti–2Cu | 0.29 | 0.26 | 1.14 | 0.41 | 0.14 | 0.34 | 0.35 | 80 | 30 | 0.45 | 0.73 | 0.33 |
| Al–10Ti–2Cu | 0.29 | 0.25 | 1.16 | 0.43 | 0.14 | 0.32 | 0.35 | 80 | 30 | 0.50 | 0.63 | 0.31 |
| Carbon steel (0.55 wt% C) | 1.30 | 1.08 | 1.21 | 0.49 | 0.62 | 1.27 | 0.29 | 205 | 80 | 0.61 | 3.92 | 2.38 |
| Carbon steel (0.55 wt% C) | 0.77 | 0.65 | 1.19 | 0.47 | 0.37 | 0.79 | 0.29 | 205 | 80 | 0.56 | 1.52 | 0.84 |
| Carbon steel (0.55 wt% C) | 0.60 | 0.57 | 1.06 | 0.27 | 0.29 | 1.11 | 0.29 | 205 | 80 | 0.18 | 2.99 | 0.54 |

**Table S3.** Tensile and compressive failure strengths (*σ*_tf_ and *σ*_cf_), elastic modulus (*ν, E, G*), critical failure strengths (*τ*_0_ and *σ*_0_), and critical energy densities for failure (*E*_c0_ and *E*_s0_) of some polycrystalline metals and alloys, as well as some engineering ceramics. Data of failure strengths and elastic modulus are collected from Refs. [S5,S19-S25]. The strengths for ceramics are the average ones. The failure strengths of crystalline alloys depend strongly on their microstructure; here the listed tensile and compressive strengths for each alloy were collected for one specific microstructure to show an example. The critical failure strengths and their ratio (*α*) for metals and alloys are calculated according to Eqs. (S1), (S8) and (S9), while for others according to Eqs. (S2)-(S4). The critical energy densities and their ratio for metals and alloys are calculated according to Eqs.(S10)-(S12), while for others according to Eqs. (S5)-(S7).

| **Materials** | ***σ*_cf_ (MPa)** | ***σ*_tf_ (MPa)** | ***σ*_cf_/*σ*_tf_** | ***α* = *τ*_0_/*σ*_0_** | ***τ*_0_**  **(MPa)** | ***σ*_0_**  **(MPa)** | ***ν*** | ***E***  **(GPa)** | ***G***  **(GPa)** | ***φ*** | ***E*_c0_**  **(MJ/m^3^)** | ***E*_s0_**  **(MJ/m^3^)** |
| --- | --- | --- | --- | --- | --- | --- | --- | --- | --- | --- | --- | --- |
| Pure Aluminum | 59 | 60 | 0.98 | 0 | 30 | - | 0.36 | 68 | 25 | 0.00 | - | 0.02 |
| Aluminum 2014-T651 | 470 | 483 | 0.97 | 0 | 242 | - | 0.33 | 72 | 28 | 0.00 | - | 1.04 |
| Titanium Ti-6Al-4V, Annealed | 970 | 950 | 1.02 | 0.17 | 475 | 2794 | 0.342 | 114 | 44 | 0.08 | 34.30 | 2.56 |
| Titanium Ti-15V-3Cr-3Al-3Sn | 1130 | 1110 | 1.00 | 0.15 | 555 | 3608 | 0.33 | 100 | 38 | 0.06 | 65.08 | 4.05 |
| Titanium Grade 3 | 450 | 440 | 1.02 | 0.17 | 220 | 1294 | 0.33 | 107 | 41 | 0.08 | 7.83 | 0.59 |
| Carbon steel (0.55 wt% C) | 321 | 319 | 1.01 | 0.10 | 159 | 1675 | 0.29 | 205 | 80 | 0.02 | 6.84 | 0.16 |
| AISI4310 steel | 1047 | 1067 | 1.02 | 0.16 | 534 | 3334 | 0.29 | 205 | 80 | 0.07 | 27.12 | 1.78 |
| Silicon Nitride, reaction sintered | 520 | 172 | 3.02 | 1.17 | 201 | 172 | 0.255 | 158 | 63 | 3.44 | 0.09 | 0.32 |
| Tungsten Carbide, WC | 1404 | 344 | 4.08 | 1.43 | 492 | 344 | 0.21 | 686 | 283 | 4.95 | 0.09 | 0.43 |
| Zirconia, transformation toughened, magnesia-stabilized | 1760 | 352 | 5.00 | 1.64 | 577 | 352 | 0.22 | 200 | 69 | 6.56 | 0.31 | 2.41 |
| Boron Nitride, hot pressed | 317 | 55 | 5.76 | 1.79 | 98 | 55 | 0.24 | 47 | 25 | 7.95 | 0.032 | 0.194 |
| Cordierite | 300 | 40 | 7.50 | 2.10 | 84 | 40 | 0.21 | 70 | 29 | 10.67 | 0.011 | 0.122 |
| Niobium Carbide | 2374 | 244 | 9.73 | 2.44 | 595 | 244 | 0.22 | 434 | 221 | 14.53 | 0.069 | 0.802 |
| Alumina, 99.9%, Al2O3 | 3000 | 300 | 10.00 | 2.48 | 744 | 300 | 0.22 | 370 | 150 | 15.01 | 0.122 | 1.845 |
| Portland Cement with Treated Silica Fume | 77 | 2 | 37.89 | 5.08 | 10.36 | 2.04 | 0.2 | 12 | 5 | 61.94 | 0.0002 | 0.0112 |

**References**

1. Qu, R. T., Eckert, J. & Zhang, Z. F. Tensile fracture criterion of metallic glass. *J. Appl. Phys*. **109**, 083544 (2011).
2. Qu, R. T., Calin, M., Eckert, J. & Zhang, Z. F. Metallic glasses: Notch insensitive materials. *Scripta Mater.* **66**, 733-736 (2012).
3. Sha, Z.-D., Pei, Q.-X., Sorkin, V., Branicio, P. S., Zhang, Y.-W. & Gao, H. On the notch sensitivity of CuZr metallic glasses. *Appl. Phys. Lett.* **103**, 081903 (2013).
4. Packard, C. E. & Schuh, C. A. Initiation of shear bands near a stress concentration in metallic glass. *Acta Mater.* **55**, 5348-5358 (2007).
5. Qu, R. T. & Zhang, Z. F. A universal fracture criterion for high-strength materials. *Sci. Rep.* **3**, 1117 (2013).
6. Liu, Z. Q., Qu, R. T. & Zhang, Z. F. Elasticity dominates strength and failure in metallic glasses. *J. Appl. Phys.* **117**, 014901 (2015).
7. Qu, R. T., Liu, Z. Q., Wang, R. F. & Zhang, Z. F. Yield strength and yield strain of metallic glasses and their correlations with glass transition temperature. *J. Alloys Compd.* **637**, 44-54 (2015).
8. Caris, J. & Lewandowski, J. J. Pressure effects on metallic glasses. *Acta Mater.* **58**, 1026-1036 (2009).
9. Inoue, A., Zhang, W., Zhang, T. & Kurosaka, K. High-strength Cu-based bulk glassy alloys in Cu-Zr-Ti and Cu-Hf-Ti ternary systems. *Acta Mater.* **49**, 2645-2652 (2001).
10. Zhang, G. Q. *et al.* Synthesis of centimeter-size Ag-doped Zr-Cu-Al metallic glasses with large plasticity. *J. Alloys Comp.* **424**, 176-178 (2006).
11. Yao, J. H., Wang, J. Q., Lu, L. & Li, Y. High tensile strength reliability in a bulk metallic glass. *Appl. Phys. Lett.* **92**, 041905-041903 (2008).
12. Inoue, A., Shen, B. & Takeuchi, A. Developments and Applications of Bulk Glassy Alloys in Late Transition Metal Base System. *Mater. Trans.* **47**, 1275-1285 (2006).
13. Stoica, M. *et al.* Strain distribution in Zr_64.13_Cu_15.75_Ni_10.12_Al_10_ bulk metallic glass investigated by in situ tensile tests under synchrotron radiation. *J. Appl. Phys.* **104**, 013522 (2008).
14. Donovan, P. E. A yield criterion for Pd_40_Ni_40_P_20_ metallic glass. *Acta Metall.* **37**, 445-456 (1989).
15. Carsley, J. E., Milligan, W. W., Zhu, X. H. & Aifantis, E. C. On the failure of pressure-sensitive plastic materials part II: Comparisons with experiments on ultra fine grained Fe-10% Cu alloys. *Scripta Mater.* **36**, 727-732 (1997).
16. Liu, J. Z., van de Walle, A., Ghosh, G. & Asta, M. Structure, energetics, and mechanical stability of Fe-Cu bcc alloys from first-principles calculations. *Phys. Rev. B* **72**,144109 (2005).
17. Cheng, S., Spencer, J. A. & Milligan, W. W. Strength and tension/compression asymmetry in nanostructured and ultrafine-grain metals. *Acta Mater.* **51**, 4505-4518 (2003).
18. Liu, Z. Q., Wang, R. F., Qu, R. T., Wang, W. H. & Zhang, Z. F. Precisely predicting and designing the elasticity of metallic glasses. *J. Appl. Phys.* **115**, 203513 (2014).
19. Yu, C. Y., Sun, P. L., Kao, P. W. & Chang, C. P. Mechanical properties of submicron-grained aluminum. *Scripta Mater.* **52**, 359-363 (2005).
20. Rodríguez-Baracaldo, R., Benito, J. & Cabrera, J. Tensile and compressive test in nanocrystalline and ultrafine carbon steel. *J. Mater. Sci.* **45**, 4796-4804 (2010).
21. Holt, J. M. T. & Ho, C. Y. *Structural Alloys Handbook*. (CINDAS/Purdue University, 1996).
22. Welsch, G., Boyer, R. & Collings, E. W. *Materials Properties Handbook: Titanium Alloys*. (ASM International, 1994).
23. Drucker, D. Plasticity theory, strength-differential(SD) phenomenon, and volume expansion in metals and plastics. *Metal. Mater. Trans. B* **4**, 667-673 (1973).
24. Bauccio, M. *ASM Engineered Materials Reference Book*. Second Edition edn, (ASM International, 1994).
25. Xu, Y. & Chung, D. D. L. Improving Silica Fume Cement by Using Silane. *Cement Concrete Res.* **30**, 1305 (2000).
